# Data supporting the rat brain sample preparation and validation assays for simultaneous determination of 8 neurotransmitters and their metabolites using liquid chromatography–tandem mass spectrometry

**DOI:** 10.1016/j.dib.2016.03.025

**Published:** 2016-03-11

**Authors:** Aneta Wojnicz, José Avendaño Ortiz, Ana I. Casas, Andiara E. Freitas, Manuela G. López, Ana Ruiz-Nuño

**Affiliations:** a Instituto-Fundación Teófilo Hernando, Facultad de Medicina, Universidad Autónoma de Madrid, Madrid, Spain; b Departamento de Farmacología y Terapéutica, Facultad de Medicina, Universidad Autónoma de Madrid, Madrid, Spain; c Servicio de Farmacología Clínica, Instituto de Investigación Sanitaria, Hospital Universitario de la Princesa, Universidad Autónoma de Madrid, Madrid, Spain; d Center of Biological Sciences, Universidade Federal de Santa Catarina, Campus Universitário, Trindade, Florianópolis, Brasil; e Department of Pharmacology, CARIM, and Maastricht Institute for Advanced Studies, Maastricht University, The Netherlands

**Keywords:** ACN, acetonitrile, AD, adrenaline, CV, coefficient of variation, DA, dopamine, EMA, European Medicine Agency, ESI, electrospray ionization, FDA, United States Food and Drug Administration, GABA, γ-aminobutyric acid, Glu, glutamic acid, 5-HIAA, 5-hydroxyindole-3 acetic acid, 5-HT, 5-hydroxytryptamine, IS, internal standard, LC–MS/MS, liquid chromatography–tandem mass spectrometry, LLOQ, lower limit of quantification, MHPG, 3-methoxy-4-hydroxy phenylglycol, NA, noradrenaline, PPT, protein precipitation, QC, quality control

## Abstract

The data presented in this article supports the rat brain sample preparation procedure previous to its injection into the liquid chromatography–tandem mass spectrometry (LC–MS/MS) system to monitor levels of adrenaline, noradrenaline, glutamic acid, γ-aminobutyric acid, dopamine, 5-hydroxytryptamine, 5-hydroxyindole acetic acid, and 3-methoxy-4-hydroxyphenylglycol. In addition, we describe the method validation assays (such as calibration curve, lower limit of quantification, precision and accuracy intra- and inter-day, selectivity, extraction recovery and matrix effect, stability, and carry-over effect) according to the United States Food and Drug Administration and European Medicine Agency to measure in one step different neurotransmitters and their metabolites. The data supplied in this article is related to the research study entitled: “Simultaneous determination of 8 neurotransmitters and their metabolite levels in rat brain using liquid chromatography in tandem with mass spectrometry: application to the murine Nrf2 model of depression” (Wojnicz et al. 2016) [1].

**Specifications table**

**Table:** 

Subject area	*Analytical Chemistry*
More specific subject area	*Biochemistry, neurotransmitters*
Type of data	*Table, figure*
How data was acquired	*MS/MS was acquired in a triple quadrupole*
Data format	*Raw mass spectra data, analyzed data and excel files (xls)*
Experimental factors	*Brain samples were homogenized in ice-cold 1.89% formic acid in water and centrifuged. Acetonitrile with 1% formic acid was added to the supernatant with internal standard for protein precipitation (4:1, v/v) and centrifuged. The supernatant was evaporated and reconstituted with mobile phase before injection**.***
Experimental features	*Rat brain was homogenized and protein precipitation as sample preparation was performed. Neurotransmitters and metabolites were determined by liquid chromatography-tandem mass spectrometry. Method validation was carried out according to the regulatory agencies.*
Data source location	*Madrid, Spain*
Data accessibility	*Data is within this article*

**Value of the data**

• Dataset of product ion mass spectra of 8 neurotransmitters and metabolites using a triple quadrupole.
• The data presented the sample preparation procedure performed to clean up the rat brain samples.
• Dataset of method validation assays according to the recommendations of United States Food and Drug Administration and European Medicine Agency.
• Rapid quantification of neurotransmitters and metabolites using this method is of potential use to diagnose neurological diseases.

## 1 Data

The following dataset includes 2 figures and 1 table that support the sample preparation and method validation assays required to monitor levels of adrenaline (AD), noradrenaline (NA), glutamic acid (Glu), γ-aminobutyric acid (GABA), dopamine (DA), 5-hydroxytryptamine (5-HT), 5-hydroxyindole acetic acid (5-HIAA), and 3-methoxy-4-hydroxyphenylglycol (MHPG) by liquid chromatography–tandem mass spectrometry (LC–MS/MS) in rat brain samples.

## 2 Experimental design, materials and methods

### 2.1 Chromatographic and mass spectrometry conditions

**Fig. 1:**
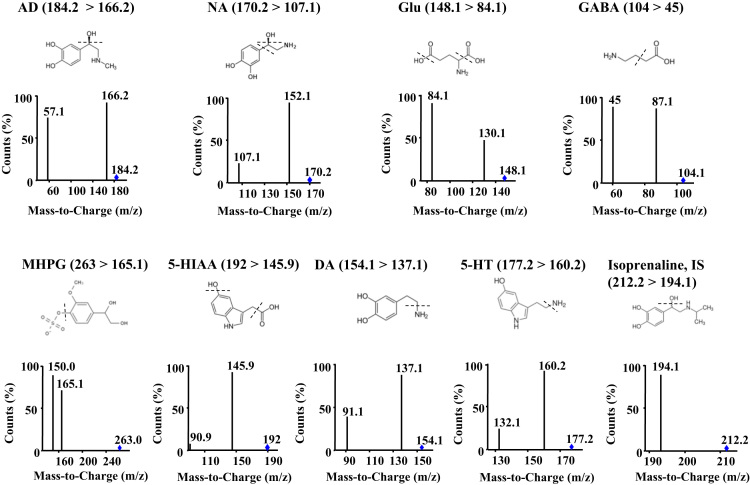
Product ion spectrums and chemical structures with the fragmentation pattern of neurotransmitters and their metabolites. The precursor ion and the product ions are shown in the figure. The quantifier multiple-reaction monitoring transition is indicated for each compound. The chemical structure with the fragmentation pattern is shown. All chromatograms have been normalized to the largest peak. Abbreviations: AD: adrenaline; NA: noradrenaline; Glu: glutamic acid; GABA: γ-amino butyric acid; DA: dopamine; 5-HT: serotonin; MHPG: 3-methoxy-4-hydroxyphenylglycol; 5-HIAA: 5-hydroxyindoleacetic acid**;** IS: internal standard.

All the chromatographic and mass spectrometry conditions are related to [1]. Here, we describe raw dataset of product ion mass spectra. Fig. 1 shows the mass spectra of the precursor ion and the product ion that were optimized and the fragmentation pattern for each analyte. The integration peak area corresponding to the transition mass to charge (*m*/*z*) of each analyte was quantified using Mass Hunter Workstation Quantitative Analysis software (Agilent Technologies, Santa Clara, Unites States).

### 2.2 Sample preparation

The data described here has been carried out in accordance with Ethics Committee of the School of Medicine-Universidad Autónoma de Madrid- and international guidelines of animal care and welfare for the experiments involving animals. Whole brain was obtained from adult male Sprague-Dawley rats (250–300 g). The rats were sacrificed by decapitation and the brain tissue was frozen immediately at –80°C. The brain samples were weighed and homogenized using Polytron PT 1200C (Kinematica, Lucerne, Switzerland) in ice-cold 1.89% formic acid in water at a concentration of 10 mL/g tissue and centrifuged at 14,000 rpm and 4°C for 40 min.

**Fig. 2:**
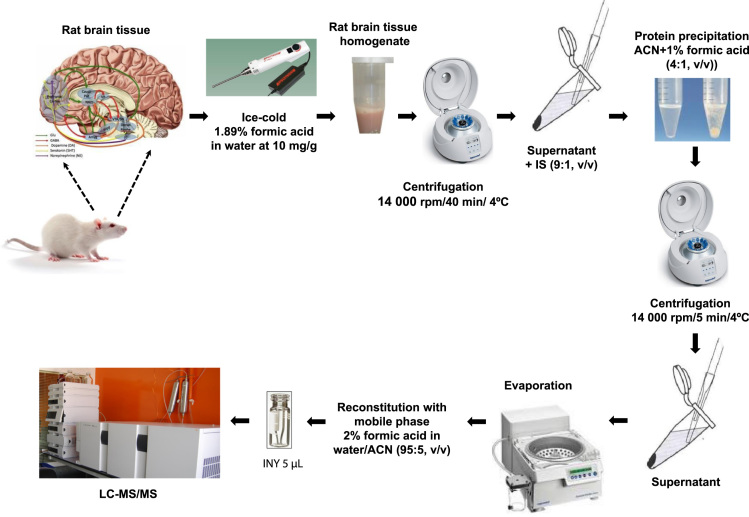
Sketch of sample preparation procedure.

The supernatant was measured and isoprenaline (internal standard**,** IS) was added to a final concentration of 500 ng/mL in a 9:1 proportion (v/v). ACN with 1% formic acid was added to the supernatant with IS for protein precipitation (PPT) in a 4:1 proportion (v/v) and centrifuged at 14,000 rpm and 4°C for 5 min. The supernatant was then evaporated to dryness using a concentrator (5301, Eppendorf, Germany) at 45°C for 1 h and 15 min. Finally, the dry residue was reconstituted with the mobile phase (0.2% formic acid in water/ACN [95:5, v/v]) with the same volume obtained before PPT. Samples were transferred to vials to be injected directly into the LC–MS/MS (Fig. 2).

Owing to the high concentrations of the endogenous analytes of GABA, Glu, DA, and 5-HT, the brain homogenate supernatant was diluted 100 times before PPT for the validation assays.

### 2.3 Assay validation procedures

The datasets of method validation assays described here are according to FDA [2] and EMA [3] recommendations. Calibration standards and quality controls (QCs) were prepare from independent dilutions of each stock solution and spiked in the brain homogenate. The concentrations of analytes in the QC solutions were calculated by using calibration curves on every validation day. Because of the presence of endogenous analytes in brain samples, the response of the blank matrix sample should be subtracted from each calibration point and QC.

#### 2.3.1 Calibration curve and lower limit of quantitation (LLOQ)

Quantitative analysis of neurotransmitters and their metabolites was performed in rat brain homogenate using isoprenaline as the IS (500 ng/mL). Eight calibration standards in concentration ranges of 0.25–200, 0.5–200, 250–20,000, 250–20,000, 0.25–200, 10–3000, 1–50, and 1–50 ng/mL were used for validation in the case of AD, NA, Glu, GABA, DA, 5-HT, 5-HIAA, and MHPG, respectively. A weighted linear or polynomial regression model adjusted for least squares was used to calculate the equation relating the area ratio of analyte versus IS to the concentration of analyte in the calibration standards (Table 1). From 3 to 5 calibration curves were analyzed for each compound. The standard curve was chosen to cover the range of the relevant endogenous concentrations expected in most rat brain tissue. At least 6 of 8 calibration standards should be less than 15% of the coefficient of variation (CV) in order to validate the calibration curve. For each point of the calibration curve, the error of accuracy and CV should be less than 15% for all calibration standards, except for the LLOQ, which was less than 20%. The LLOQ response of the analyte should be at least 5 times higher than the blank response.

#### 2.3.2 Precision and accuracy

Critical factors for measurement of the reproducibility of the assay are precision, which is defined as the repeatability of the assay, and accuracy, which is the closeness to the true value of the value obtained by the method. The precision and accuracy of the dataset were assessed by analyzing 5 replicates per 4 concentration levels in a single analytical run, namely, LLOQ and low, medium, and high QCs, which covered the calibration curve range. The intra-day precision and accuracy were evaluated by analyzing 5 replicates of each QC level on a single day. The inter-day variation was evaluated by injecting each QC sample in 5 replicates over 3 analytical runs from at least 3 different days.

Precision is expressed as the CV (%). Accuracy was defined as the percentage difference between the theoretical and the measured value according to the following equation:

$$\frac{\mathrm{Accuracy}\left(\%\right)\;=\;\left[{\mathrm{concentration}}_{\mathrm{measured}}{–\text{concentration}}_{\mathrm{theoretical}}\right]}{\left[{\mathrm{concentration}}_{\mathrm{theoretical}}\right]\times 100\%}$$

To validate precision and accuracy, the error must be less than 15% for all QCs except the LLOQ, which must be within 20% of the true value.

#### 2.3.3 Selectivity

The selectivity of the method is the ability to differentiate the analytes of interest and IS from endogenous or other components in the matrix. Selectivity was tested in 6 different lots of rat brain homogenates with IS (zero matrix sample) or without IS (blank matrix sample), which were then individually analyzed for interference. Absence of interfering components should be accepted where the response is less than 5 times (20%) the LLOQ for the analyte and 20 times (5%) for the IS. Because of the presence of endogenous compounds, we compared the response of LLOQ after blank matrix sample subtraction with blank sample response (mobile phase).

#### 2.3.4 Extraction recovery and matrix effect

The relative recovery to its IS is evaluated as the ratio of compound concentrations in brain homogenate following PPT to the same concentration dissolved directly in reconstitution solution. Three repetitions of low and high QCs for neurotransmitters and their metabolites were analyzed in 3 different batches of rat brain homogenates. Therefore, recovery refers to the efficiency of extraction of an analysis method. Recovery of the analyte need not be 100% to be adequate, but the extent of recovery of QC samples should be precise, reliable, and reproducible.

The relative matrix effect of rat brain homogenate was measured through the addition of a known concentration of analyte with its IS to a rat brain homogenate that had undergone PPT. This response is compared with the addition of the same amount of analyte and IS to a final reconstitution solution. This time, 6 repetitions per concentration level were analyzed in 6 different lots of rat brain homogenate at low and high QCs for neurotransmitters and their metabolites. To validate the matrix effect, the CV should not be greater than 15% for all the QCs.

#### 2.3.5 Stability

We evaluated the stability assays at 2 concentration levels (low and high QCs), as follows: (1) after 24 h at 23°C in the autosampler; (2) after 24 h at 4°C in the fridge; (3) after 3 cycles of freeze–thaw in the freezer at –80°C. For all assays, 3 replicates of low and high QCs for all the analytes were performed and analyzed according to the criteria of the FDA and the EMA. The stability was considered acceptable if it was within a CV of less than 15% for all the QCs used. In this way, we guaranteed sample preparation, the analytical process, and the storage conditions.

#### 2.3.6 Carry-over

Carry-over is the presence of an analyte signal in a blank sample after the analysis of samples with a high analyte concentration. Transfer should be minimal. Carry-over should be assessed by injecting blank samples after a high concentration sample or calibration standard at the upper limit of quantification. Carry-over in the blank sample following the high concentration standard should not exceed 20% of the LLOQ. Between injections, the needle was washed with water and methanol (50:50) solution to prevent carry-over.

## Figures and Tables

**Table 1:** Concentration ranges, weighted regression models adjusted, correlation coefficient, and lower limits of quantification for each compound.

**Compound**	**Equation**	**Linear range**	**Correlation**	**LLOQ**

		**(ng/mL)**	**Coefficient mean±SD**	**(ng/mL)**

AD	Polynomial 3rd order	0.25–185	1.0000±0.0000	0.25
NA	Linear*	0.5–200	0.9996±0.0004	0.50
Glu	Polynomial 3rd order	250–20,000	0.9999±0.0001	250
GABA	Polynomial 3rd order	250–20,000	1.0000±0.0000	250
DA	Polynomial 3rd order	0.25–200	1.0000±0.0000	0.25
5-HT	Linear*	10–3000	0.9948±0.0040	10
MHPG	Polynomial 3rd order	1–50	0.9993±0.0002	1
5-HIAA	Linear	1–50	0.9936±0.0023	1

Abbreviations: AD: adrenaline; NA: noradrenaline; Glu: glutamic acid; GABA: γ-amino butyric acid; DA: dopamine; 5-HT: serotonin; MHPG: 3-methoxy-4-hydroxyphenylglycol; 5-HIAA: 5-hydroxyindoleacetic acid.
